# New Insights into the Mechanisms of Toxicity of Aging Microplastics

**DOI:** 10.3390/toxics12100726

**Published:** 2024-10-08

**Authors:** Victor Pavlovich Chelomin, Aleksandra Anatolyevna Istomina, Andrey Alexandrovich Mazur, Valentina Vladimirovna Slobodskova, Avianna Fayazovna Zhukovskaya, Nadezhda Vladimirovna Dovzhenko

**Affiliations:** V.I.l’ichev Pacific Oceanological Institute, Far Eastern Branch, Russian Academy of Sciences, Vladivostok 690041, Russia

**Keywords:** polypropylene, UV irradiation, oxidative stress, mussel, reactive oxygen species

## Abstract

Nowadays, synthetic polymer (plastic) particles are ubiquitous in the environment. It is known that for several decades microplastics (MPs) have been accumulating in the World Ocean, becoming available to a large variety of marine organisms. Particularly alarming is the accumulation of aging plastic particles, as the degradation processes of such particles increase their toxicity. The diverse display of negative properties of aging MPs and its effect on biota are still poorly understood. In this study, in vitro experiments modeling the interaction of pristine and UV-irradiated aging polypropylene (PP) fragments with hemocytes and mitochondria of bivalve mollusks *Mytilus* sp. were performed. The appearance of free radicals in the environment was recorded by spectral characteristics of indicator dyes—methylene blue (MB) and nitroblue tetrazolium (NBT). It was found that due to photooxidation, aging PP fragments sorbed more than threefold MB on their modified surface compared to pristine samples of this polymer. Using NBT, the formation of reactive oxygen species in seawater in the presence of pristine and photoactivated PP was recorded. It was also found that photodegraded PP fragments largely stimulated the development of lipid peroxidation processes in mitochondrial membranes and reduced the stability of hemocyte lysosome membranes compared to pristine PP fragments. In general, the results obtained concretize and supplement with experimental data the previously stated hypothesis of toxicity of aging MPs.

## 1. Introduction

In recent times, it has become evident that synthetic polymers (plastics) are ubiquitous in the environment and have penetrated all ecosystems. Due to the threat of mass distribution of plastic and its impact on the stability of ecosystems, the relevance of research on the relationship between these polymers and biota has sharply increased. This problem is especially acute as plastic products are exposed to physicochemical and biological degradation in the environment and break down into micro-sized particles (microplastics), becoming accessible to various living organisms [1,2,3,4]. This trend is most clearly manifested in the waters of the ocean. An analysis of literature data shows that microplastics (MPs) have been accumulating in the ocean for several decades, distributing in water and sediments. At present, particles of different types of MPs are found in representatives of a wide range of marine fauna species, from the primary trophic link (small plankton) to large marine animals [1,4,5,6,7,8], causing justified concern among marine ecologists.

From a chemistry point of view, synthetic polymers are not active. Nevertheless, a subject of special attention to ecotoxicologists has become the ability of MPs to enter and accumulate in biological systems of different levels of organization, due to the development of toxic processes [9,10,11,12,13,14,15]. These concerns are based on numerous experimental studies demonstrating the damaging effect of various polymers particles, which manifest at different levels of the organism, from molecular to behavioral reactions [14,16,17,18,19,20,21,22,23,24,25]. This tendency is clearly shown when assessing the toxicity of aging polymers. A number of papers have presented quite convincing evidence that such degraded polymers strongly affect various cellular processes [26,27,28,29,30,31,32,33]. Additionally, in recent years, a growing number of publications indicate an increase in the toxic properties of polymers in which oxidative degradation processes have been initiated [3,24,34,35,36,37]. Since oxidative degradation processes occurring in aging plastics change the physical properties and chemical structure of polymers [38,39,40,41,42], it is logical to assume that they are responsible for the intensification of toxic properties. Ecotoxicologists consider this situation particularly important because in the real marine environment, hydrobionts interact with aging MPs undergoing abiotic and biotic degradation. In order to assess the impact of MPs on biological systems of different levels of organization and to learn how to predict the consequences of the presence of MPs in the environment, it is important to identify the mechanisms of their interaction with biological structures at the molecular level.

The reasons for the negative properties of aged MPs are still not clear. Nevertheless, as experimental evidence accumulates, an increasing number of authors are inclined to believe that the induction of reactive oxygen species (ROS), leading to the development of oxidative stress (OS) processes, underlies these numerous changes of toxic effect [23,25,43,44,45,46,47]. However, despite significant progress in this study, the question of how chemically inert synthetic polymers contribute to the induction of OS processes remains open.

Earlier, in our paper [37], we used the mechanisms of physicochemical processes of synthetic polymers degradation due to free-radical reactions [48,49,50,51,52], and suggested that UV-irradiated MPs could be a source of free radicals and induce prooxidative processes in biological systems.

To confirm or refute this hypothesis, the authors conducted a series of laboratory in vitro experiments modeling the interaction of pristine and artificially aged (UV irradiation) polypropylene (PP) fragments with biological structures (hemocytes and mitochondria of mollusks). During these experiments, it was evaluated the induction of prooxidative reactions by the formation of lipid peroxidation products in mitochondrial membranes (thiobarbituric acid (TBA) reactive products) and the hemocyte lysosome membrane stability. The appearance of free radicals in the medium was recorded by spectral characteristics of indicator dyes—methylene blue (MB) and nitroblue tetrazolium (NBT).

## 2. Materials and Methods

### 2.1. Characteristics of Microplastic and Dyes

The plastic polymers used in the experiment came from commercially available polypropylene rope, which chemical composition was determined by FTIR spectroscopy. PP fragments (2 mm long and 1 mm wide) used in the experiment were obtained by cutting unused rope manufactured by Trusco Nakayama Corporation (Cat No. PP-4300) (Osaka, Japan). The production technology and exact chemical composition of the rope are not specified by the manufacturer. UV irradiation of plastic fragments was carried out in a Petri dish using a lamp Supratec HTC 400-241 (Osram, Munich, Germany) with 460 W power for 120 h. The distance from the lamp to the surface of plastic particles was 3 cm.

The following dyes were used in the experiments: nitrotetrazolium blue chloride 98% (NBT) Sigma-aldrich; methylene blue 2% solution, Ecolab; Neutral red, >90%, high purity, VWR international, LLC Amresco, LLC (Solon, OH, USA).

### 2.2. Fourier-Transform Infrared (FTIR) Spectroscopy 

FTIR spectra were acquired using an IRAffinity-1S (Shimadzu, Kyoto, Japan) equipped with an attachment for frustrated total internal reflection (wavenumber range of 4000–400 cm^−1^, 32 scans per spectrum, spectral resolution of 4 cm^−1^). The background was measured with the same settings against air. The obtained spectra were processed using LabSolutions IR 2.27 software (Shimadzu, Kyoto, Japan). The content of functional groups in all PP samples was calculated using the following indices: carbonyl index (CI) [38], hydroxyl index (HI), and carboxyl index (COI) [53]. The spectra were preliminarily normalized to the reference peak at 2950 cm^−1^.

### 2.3. In Vitro Experiment: MPs and Dyes

#### 2.3.1. MPs and Nitroblue Tetrazolium (NBT)

A total of 15 mg of pristine and aging MPs were incubated for 2 h at 4 °C under constant stirring in 1 mL TBS buffer (0.05 M Tris-HCl buffer, pH 7.6, containing 2% NaCl) containing 1 mg of NBT using a thermo-shaker (Biosan) in the dark. After incubation, the buffer was removed, and the MPs were washed with TBS buffer to remove free NBT. Sorbed NBT was extracted with 1 mL of a mixture of 2M KOH in DMSO. After 30 min, the optical density at 620 nm was measured [54].

#### 2.3.2. MPs and Methylene Blue (MB)

To use the cationic dye MB as an indicator of free-radical processes, the kinetics of changes in UV–VIS spectra under conditions of ROS formation induced by Fenton reagents was recorded. The reaction mixture in a volume of 4 mL contained 15.5 μmol MB and Fenton reagents: 10 μL FeSO_4_ (0.250 mM) and 10 μL H_2_O_2_ (0.250 mM). The degradation process (discoloration) of the MB dye due to ROS attack was recorded by changing the ratio of two basic peaks (λ660/λ290). A total of 50 mg of pristine and UV-irradiated MPs were incubated under constant stirring at 20 °C in the dark for 5 h and 3 days in 5 mL of 31 μmol MB solution. The amount of MB sorbed by MPs was calculated according to the formula:

$$Q=\frac{\left(C_{0}-C_{x}\right)*V}{S}$$

where C_0_ and C_x_—concentrations of MB before and after the equilibrium state, V—volume of the reaction mixture, and S—surface area of MPs (cm^2^).

### 2.4. Biological Material

*Mytilus* sp. was collected after the spawning period in the waters of the Alekseev Bay in the Sea of Japan (42°59′ N; 131°43′ E). The isolated gills were frozen in liquid nitrogen and stored for no more than 1 month before analysis. All procedures in the present study, as well as the mollusk disposal methods, were approved by the Commission on Bioethics at the V.I.l’ichev Pacific Oceanological Institute, Far Eastern Branch of the Russian Academy of Sciences (protocol No.16 and date of approval 15 April 2021), Vladivostok, Russia.

#### 2.4.1. Mitochondria Isolation

Gills were homogenized on ice (1:5, weight/volume). Mitochondria were isolated in 0.5 M NaCl in a 0.05 M Tris-HCl (pH 7.5) medium containing 0.25 M sucrose, 1 mM EDTA, and 0.1 mM PMSF. The medium for homogenization was pre-blown with argon. The homogenate was centrifuged at 1000× *g* for 12 min to remove large residual cells and nuclei. The resulting supernatant was centrifuged at 12,000× *g* for 30 min. The mitochondria were washed from sucrose 3 times in 0.5 M NaCl in 0.05 M Tris-HCl (pH 7.5).

#### 2.4.2. Hemocytes Isolation 

Hemolymph (approximately 0.1 mL) was obtained from the anterior adductor muscle of *Mytilus* sp. using a 1 mL hypodermic syringe (the ratio of the hemolymph and the filtered seawater in the syringe was 1:1). Hemolymph from 10 to 15 animals was pooled (7 × 10^5^ cells/mL) and used in the experiment.

### 2.5. Experiment In Vitro: The Effect of MPs on Biological Material

#### 2.5.1. Effect of Microplastics MPs on Mitochondria

Mitochondria were obtained from 0.6 g *Mytilus* sp. gills. Pristine and aging MPs (5, 12.5 and 25 mg) were incubated in 1 mL of mitochondria suspension for 20 h at 20 °C (repeated three times for each plastic suspension) under constant stirring using programmable rotary mixer Multi Bio RS-24. The formation of lipid peroxidation products was determined by color reaction with 2-thiobarbituric acid [55]. Protein concentration was determined by a modified Lowry’s method [56]. Measurements were performed on a UV-1650PC spectrophotometer (Shimadzu).

#### 2.5.2. Effect of MPs on Hemocytes

A total of 1 mL hemocytes were incubated for 2 h at 4 °C under constant stirring using thermo-shaker (Biosan) with 15 mg PP (pristine and UV-irradiated). 

Detection of superoxide anions (O_2_**^·−^**) in hemocytes of mussels were detected intracellularly by a modification of a previously described method [54], with the use of nitroblue tetrazolium (NBT). The modification consisted only in replacing the ALS buffer with filtered seawater.

Uptake of the neutral red dye: the method is based on the ability of healthy hemocyte lysosomes to retain neutral red (NR) dye [57,58]. A total of 1.5 mL of hemolymph was incubated with 15 mg pristine and aging PP for 1 h. Then a 1:1 working solution of neutral red was added to the plastic-free hemolymph and incubated for 20 min. After centrifugation at 1200 rpm for 10 min, the cell precipitate was washed with 1 mL of TBS buffer (0.05 M Tris-HCl buffer, pH 7.6, containing 2% NaCl). For dye extraction, 1 mL of a mixture of 1% acetic acid and 50% ethanol was added to the cell sediment and was incubated for 15 min. All procedures were performed at 4 °C. Absorbance was measured at 540 nm. Cell viability was evaluated as % survival from control.

NR stock solution (100 mM) was prepared by dissolving 28.8 mg of dye powder in 1 mL of DMSO [59]. The dye working solution was prepared by dissolving 10μLof stock NR in 5 mL of filtered seawater.

### 2.6. Statistics

Statistical processing of the results was performed using Statistica 7. Each experiment was repeated three times. The Mann–Whitney’s U test for non-parametric variables was used to assess reliability of parameter changes. Significance was established at *p* < 0.05.

## 3. Results

### 3.1. Characterization of Pristine and Aging MPs

#### 3.1.1. FTIR Spectroscopy

**Figure 1:**
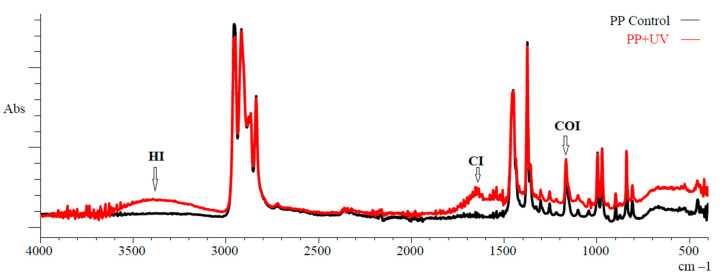
FTIR spectra of polypropylene (PP) after 120 h UV irradiation. CI, carbonyl index; HI, hydroxyl index; COI, carbon-oxygen index.

The MPs used in our study as model plastic were subjected in vitro to UV irradiation to initiate oxidative degradation of the polymer structure. Fourier transform infrared spectroscopy was used to characterize the chemical changes. Figure 1 shows the spectra of surface functional groups of PP before and after UV exposure under our experimental conditions.

According to FTIR data, the initial PP fragments had spectral bands of 3000–2800 cm^−1^ and 1450–1370 cm^−1^, characteristic of this polymer, related to stretching and bending vibrations of the C−H bond of alkanes (polyolefins), respectively. Significant changes are observed in the spectrum of PP after continuous UV irradiation for 120 h, due to the appearance of new peaks in the frequency ranges of 3600–3200 cm^−1^ and 1750–1650 cm^−1^. These spectral peaks are related to the stretching of OH bond (hydroxyl group) and valence C=O bond (carbonyl groups) oscillation, respectively. Carbonyl (CI), carboxyl (COI), and hydroxyl (HI) indexes were used to quantify the degree of oxidation of polymer chains of PP after UV irradiation. The calculated index values for pristine and UV-irradiated PP are summarized in Table 1. A reliable 2-fold increase of the CI index was observed.

#### 3.1.2. Sorption of Methylene Blue Dye by MPs 

To evaluate the changes that occurred on the surface of the PP fragments because of UV irradiation, experiments on the adsorption of the cationic MB dye were conducted. These experiments showed that artificially aging PP rapidly (~3-fold) increased the amount of sorbed dye compared to pristine polymer samples. Under equilibrium conditions after 5 h, fragments of pristine PP sorbed no more than 0.16 μmol MB/cm^2^, whereas the UV-irradiated PP bound more than 0.50 μmol MB/cm^2^ (Table 2).

#### 3.1.3. Registration of ROS Generated by MPs in Seawater

Two specific indicator dyes, MB and NBT, were used to record the generation of ROS in the seawater in the presence of both forms of PP. These dyes show high sensitivity to free radicals, changing their spectral characteristics.

**Figure 2:**
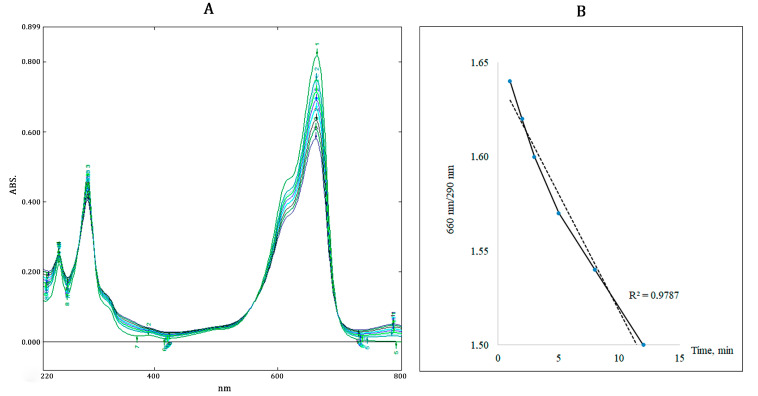
(**A**) Variations in the UV–VIS spectrum of MB induced by Fenton reagents, (**B**) linear discoloration of MB during the Fenton reaction over time.

The kinetics of changes in UV–VIS spectra under conditions of ROS generation induced by Fenton’s reagents is presented in Figure 2A. The results of these experiments revealed a significant decrease in optical density in the visible region (λ 660 nm) and a weak decrease in the UV region (λ 290 nm) of the dye spectrum. This indicates that the reactive oxygen species (mainly −OH^•^) generated by Fenton reactions cause the destruction of the chromophore group in the dye molecule. The process of destruction (discoloration) of the MB dye because of the ROS attack, shown in Figure 2B as a change in the ratio of the two main peaks (λ660/λ290) in time, has a linear character and proceeds relatively actively.

These characteristic spectral changes were revealed in the dye after exposure to pristine and aging PP fragments. Calculations based on the comparison of the ratios of the main peaks (λ660/λ290) before and after exposure show that in our experimental conditions the fraction of MB dye decolorized within 5 h with the participation of pristine PP fragments does not exceed 2.9%, whereas UV-irradiated PP fragments destroyed more than 16% of the dye during the same time. After longer exposure (72 h) with photoactivated PP fragments, the level of decolorized dye exceeded 25%.

A similar trend was observed in the experiments using the ROS-sensitive (mainly O_2_^•−^) NBT dye. Exposing the NBT in seawater with pristine and aging PP fragments, the authors registered the production of formazanes (λ − 620 nm), which indicates the appearance of O_2_^•−^ in the medium. Moreover, in the case of UV-irradiated PP fragments, this process was more intensive (by 22.8%) compared to pristine PP.

### 3.2. Pro-Oxidative Processes Induced by MPs Samples 

#### 3.2.1. Lipid Peroxidation of Mitochondrial Membranes

**Figure 3:**
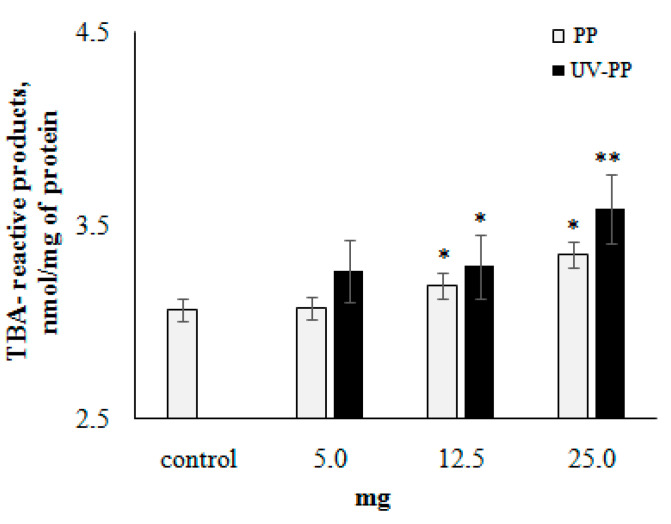
Mitochondrial lipid peroxidation after exposure to pristine and UV-irradiated PP. Results are given as means and standard errors of free measurements per experimental group. *—indicates significant differences with respect to control, **—control and exposure to pristine PP (*p* < 0.05).

The effect of pristine and aging PP fragments on the development of lipid peroxidation (LPO) processes in mitochondrial membranes was investigated in a model in vitro. The results of these studies, summarized in Figure 3, show that in the presence of plastic samples, the content of LPO products increases significantly in mitochondrial membranes in a dose-dependent manner. In the presented experiments, UV-irradiated PP fragments have a greater influence on LPO activation compared to pristine samples of this polymer.

#### 3.2.2. Effect of MPs on the Stability of Lysosome Membranes in Mussel Hemocytes

**Figure 4:**
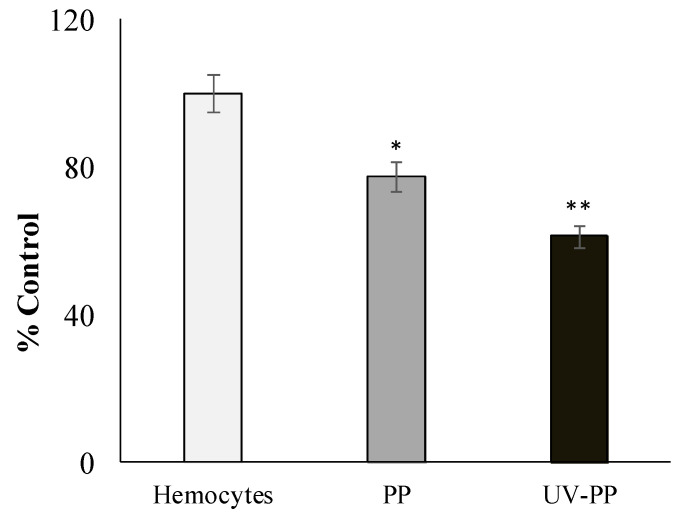
Retention of NR by hemocytes lysosomes (% control) after exposure to pristine and UV-irradiated PP. Results are given as means and standard errors of free measurements per experimental group. *—indicates significant differences (*p* < 0.05) with respect to control, **—control and exposure to pristine PP (*p* < 0.05).

Lysosomes of mollusk hemocytes are very sensitive to ROS, which destabilize and change membrane permeability by oxidizing the lipid matrix. Neutral red (NR), used in cytochemical test dye shows that in lysosomes of mussel hemocytes exposed with pristine and UV-irradiated PP fragments, the ability to retain NR dye is significantly reduced, which indicates destabilization of lysosome membranes (Figure 4).

At the same time, on the basis of the data presented in Figure 4, it can be stated that photoactivated PP fragments have a greater destabilizing effect on hemocyte membranes compared to pristine samples.

#### 3.2.3. Formation of ROS during MPs Exposure to Hemocytes

**Figure 5:**
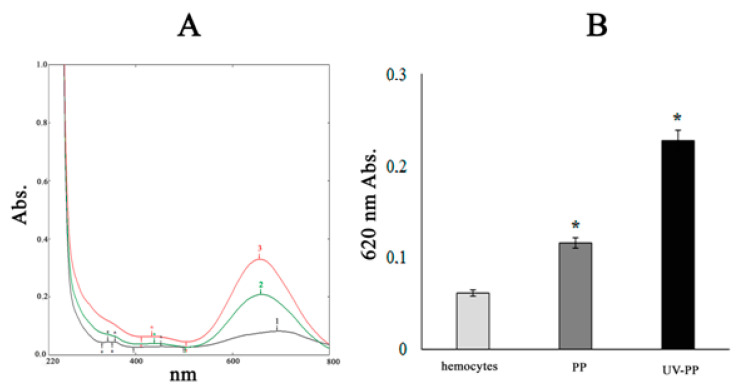
The formazane production (**A**,**B**) at 620 nm in mussel hemocytes (1—control, 2, 3—after exposure to pristine and UV-irradiated PP, accordingly). Results are given as means and standard errors of free measurements per experimental group. *—indicates significant differences (*p* < 0.05) with respect to control.

Under physiological conditions, the NBT dye interacts with ROS leading to production of formazanes, which are recorded spectrophotometrically at λ − 620 nm. This absorption peak in the NBT spectra (Figure 5A) during the exposure of both experimental plastic samples in mollusk hemocytes reflects the level of ROS formation. These experiments show that in the presence of photoactivated PP fragments, 2-fold more ROS are generated in mussel hemocytes as compared to exposure to pristine PP sample (Figure 5B).

## 4. Discussion

Unlike chemical compounds that to different degrees can interact with a variety of functional biostructures, synthetic polymer particles are chemically inert, making it very difficult to understand their biological activity. Therefore, there are concerns in the literature that fragments of plastics, which were applied during polymer production or sorbed during operation and migration through various contaminated environmental areas, may release potentially toxic chemicals into the marine environment or directly into the internal environment of the body (when ingested) [25,60,61,62,63,64,65]. However, with the accumulation of evidence, it is becoming increasingly obvious that in the real marine environment the hydrobionts interact with modified fragments of plastics that are characterized by various degrees of physicochemical degradation.

Synthetic polymers are constantly affected by a variety of abiotic (temperature, atmospheric and dissolved O_2_, UV sunlight, dissolved salts, polluting organic and inorganic substances, wind and mechanical impact, etc.) and biotic (microbes, phytoplankton, zooplankton, and invertebrate larvae) factors in the marine environment. These impacts initiate complex destructive aging processes in polymers, accompanied by changes in chemical and physical structure, called “weathering” of polymers. Given that UV light and dissolved O_2_ play a major role in plastic weathering, this process has been successfully modeled in vitro. It helps to accelerate degradation processes and to study the physical and chemical properties of aging polymers [8,39,44,66,67,68,69,70,71]. 

The initial stages of this complex destructive process are clearly demonstrated in our experiments during UV irradiation of PP samples (Figure 1), which are characterized by the highest photooxidation rate among polyolefin polymers [72]. Oxidative aging of PP during artificial UV irradiation is confirmed by the formation of new ones and modifications to existing absorption bands in IR spectra, mainly in the frequency ranges corresponding to hydroxyl (O−H, 3600–3200 cm^−1^), carbonyl (C=O, 1750–1600 cm^−1^), and carboxyl (C−O, 1200–1050 cm^−1^) groups [48,73,74,75,76]. The appearance of a broad absorption band for the OH group (3600–3200 cm^−1^) in the IR spectra of PP after UV irradiation indicates the formation of free (about 3550 cm^−1^) and associated (about 3410 cm^−1^) hydroperoxides (ROOH) in the structure of polymer chains [77]. In addition, strong changes in the IR spectra are detected in the carbonyl area, with a broad absorption band (1750–1600 cm^−1^) reflecting the formation of several products (ketoacids, aldehydes, and ketones), among which the dominant absorption band is (1734 cm^−1^) corresponding to the carbonyl group of esters [38]. As shown by Duan et al. [50], the oxidative degradation of PP induced by UV irradiation leads to the carbonyl groups formation in the aliphatic due to −H abstraction and C−C bond breakage. Also, as a result of photooxidation, a new absorption peak (1653 cm^−1^) has been reported in the polymer chain structure of PP, indicating the formation of a vinyl group [73,76]. The C=C bond formation may occur mainly because of the C−C breakdown of the main chain carbon bond initiated by a substituent (−C−R) and the breakdown of the C−H bond [73,76].

From the comparative analysis of the IR spectra (Figure 1), it is evident that UV induced oxidation of the PP fragments leads to changes in the range of 1200–1050 cm^−1^, corresponding to the spectral range of the carboxyl bond (C−O), which also indicates the accumulation of different oxidative degradation products of the polymer [53]. On the basis of the spectral characteristics and calculated degradation indexes (Table 1), especially CI and HI, it can be stated that UV irradiation initiated the development of oxidative degradation processes, leading to a significant change in the chemical composition of PP surface chains. First, this is manifested in the oxygen-containing functional groups appearing in the structure of artificially aging PP, which significantly affect the physicochemical characteristics of polymers. in particular, they enhance hydrophilicity and sorption properties [8,68,70,71,78,79,80,81]. Our experiments with the sorption of the cationic dye MB (methylene blue) serve as a clear example of these changes. It turned out that due to photooxidation, the UV-irradiated PP fragments sorbed more than 3-fold more dye on their modified surface compared to pristine samples of this polymer (0.50 μmol MB/cm^2^ compared to 0.16 μmol MB/cm^2^). This is due to the introduction of oxygen-containing groups, such as keto- and hydroxy groups, into the polymer structure, which have a negative charge at seawater pH, resulting in increased binding of the cationic dye due to electrostatic forces [82,83].

It is logical to assume that this complex of chemical and physical changes in polymers during the aging process enhances the amplification of negative effects on biological systems. To clarify this, we used in vitro model experiments, which are the most adequate and necessary approach for revealing these effects, especially indispensable for elucidating the mechanisms of biological activity of xenobiotics such as synthetic polymers. As shown by our model experiments, at the subcellular and cellular levels, UV-irradiation PP fragments largely stimulated the development of lipid peroxidation processes in mitochondrial membranes and decreased the stability of hemocyte lysosome membranes compared to pristine PP fragments (Figure 3 and Figure 4). The results of these experiments deserve special attention, as they indicate the participation of ROS in the impact of PP fragments on biological structures. The lipid matrix of mitochondrial membranes, characterized by a high degree of unsaturated fatty acids, is a sensitive target for ROS attacks, which result in the development of free-radical processes leading to the formation of TBA-reactive products. To explain the reasons for the negative effect of PP fragments on mussel hemocytes, it should be taken into account that the leading factor of lysosome membrane destabilization is ROS [84], which can be generated by direct and indirect mechanisms induced by exposure to MPs [16,85,86]. From this point of view, the results of experiments with nitroblue tetrazolium (NBT) dye (Figure 5), which is sensitive to ROS, provide evidence for the direct involvement of ROS in the destructive processes that occur when plastics are exposed to biological systems. It is noteworthy that in these experiments, the UV-irradiation PP fragments induce the formation of ROS in hemocytes almost 2-fold more intensively as compared to pristine fragments of this polymer.

Despite the model character of our experiments, the results obtained allow us to emphasize two facts. In the first place, these results complement the few experimental studies and agree well with the gaining popularity of the point of view that polymers degraded as a result of aging processes are more toxic than pristine samples of plastics [3,24,34,35,36,37]. Secondly, the character of these effects suggests that the molecular basis of the biochemical shifts obtained by us, and cited in the literature, is an increase in the generation of ROS. According to a number of authors, the toxicity of various MPs is determined by their ability to induce the formation of ROS in biological systems, which leads to the development of oxidative stress (OS) [23,25,43,44,45,46,47,51,87,88].

At present, the OS processes initiated by the penetration of MPs into living organisms are widely and extensively covered in the literature, whereas the mechanisms of ROS generation are still not clear and are hypothetical in character. Given the relatively hydrophobic nature of synthetic polymers, it has been suggested that MPs may to some extent disorganize the receptor-signaling system of the cell membrane and initiate the activation of prooxidative processes [37,89,90]. A clear example is the paper by Mazur et al. [91], in which the authors show that polystyrene microspheres, without penetrating inside the sperm of the sea urchin *Scaphechinus mirabilis*, cause an increase in the oxidative destruction of DNA molecules. As support for this mechanism, we can cite the results of the studies indicating the membranotropic character of MPs. Using artificial lipid membranes as an example, the authors [92,93] showed that micro- and nano-sized plastic particles cause physical changes and damage in the lipid matrix (bilayer stretching, viscosity changes), which can lead to serious disturbances in membrane functions and cellular mechanisms. In addition, considering that such particles overcome membrane barriers and penetrate into cells, Das et al., [88] proposed another mechanism. They believe that polymer particles can accumulate in mitochondria and cause a complex set of damages (disruption of the electron-transport chain, destabilization, and depolarization of membranes), which lead to the formation of ROS and subsequent oxidative destruction of cellular biostructures.

We do not question the validity of these assumptions; however, on their basis, it is difficult to explain the reasons for the enhancement of biological activity of aging MPs. In searching for explanations for the enhancement of ROS formation and the prooxidative effects of UV-irradiation PP fragments obtained in our experiments, we consider it reasonable to address to the known mechanisms of physical and chemical degradation of synthetic polymers. There is a point of view that polymer aging in natural and laboratory conditions proceeds through free-radical mechanism [48,49,50,51,52,94,95]. Under UV exposure and in the presence of O_2_ in polymers, including PP, mass formation of radicals is induced in polymers, which, spreading in polymer chains, induce a cascade of autocatalytic reactions. These complex reactions lead to the rupture of polymer chains and the formation of various oxidized groups, which the authors have recorded by IR spectrometry when characterizing the photodegradation of PP. Ecotoxicologists consider that, when analyzing the main mechanisms of UV-induced aging processes, it is important to emphasize that the photochemical transformation of polymers goes along with the formation of several environmentally persistent free radicals and many mobile ROS, the appearance of which was recorded in the micro-PS suspension [94]. According to the data of Jeon et al., [95], aging PP fragments under artificial conditions generated 1.5-fold more ROS on the polymer surface than pristine samples of plastic. Another example that deserves special attention is the paper by Ding et al., [49]. In the process of photooxidation of PS, the authors registered the appearance of ROS in the medium, which accelerated the photolysis of the antibiotic tetracycline.

In this respect, the results of our experiments using MB and NBT dyes sensitive to ROS [54,96,97] agree well and complement these studies. The character of spectral changes of both dyes confirms the ROS formation (O_2_**^·–^** and OH**^·^**) in the presence of PP polymer fragments. Also, simple calculations based on these spectral changes indicate that UV-irradiated PP fragments generate more ROS compared to pristine samples of this polymer.

These results suggest that due to photochemical transformation of PP chains, ROS are formed on the polymer surface in the oxygen diffusion zone, which, in contact with mitochondria and hemocytes, causes the activation of lipid peroxidation of mitochondrial membranes and destabilization of lysosome membranes. Despite the relatively small degree of oxidative destruction of the lipid matrix in our experiments, we believe that in vivo any source of reactive molecules, presents an additional risk of the spread of destructive oxidative processes and the development of pathological consequences.

## 5. Conclusions

In general, the obtained results concretize and provide experimental data to the previously stated hypothesis of toxicity of aging plastic fragments. Within the framework of this hypothesis, we believe that the mechanism of biological activity of synthetic polymers, especially aging fragments, may be associated not only with their hydrophobicity but also with their ability to form low-molecular free radicals (in the process of physicochemical transformation), which are initiators of OS development in living organisms. In other words, the proposed hypothesis is based on the probability of transferring free-radical reactions from surface polymer structures to biological structures, which is obviously mediated by ROS. Regardless of the specific mechanism of toxicity of synthetic polymers, it is necessary to emphasize the following. Currently, plastic garbage accumulation in the biosphere, increasing in particular in the marine environment, undergoes serious physical and chemical changes over time. Moreover, not only the size (macro-, micro- and nano-size), shape, and physical state (crystallinity, brittleness, porosity), but also, most importantly, the chemical composition of polymers, changes. In this regard, it is important to realize that as plastics age over time, the toxic potential of plastics in the biosphere increases.

## Figures and Tables

**Table 1:** Indexes of polypropylene degradation after UV irradiation for 120 h.

	CI	HI	COI
Pristine PP	0.23 ± 0.23	0.05 ± 0.01	0.32 ± 0.01
Aging PP	0.52 ± 0.35 *	0.06 ± 0.01	0.34 ± 0.04

Note: data is presented as mean value ± standard deviation (*n* = 8); *—significant differences vs. control (*p* < 0.05).

**Table 2:** Oxidation-reduction properties and adsorption of methylene blue (MB) upon interaction with MPs for 5 h.

	Adsorption, µmol MB/cm^2^	Oxidation/Reduction, % of Control
Pristine PP	0.16	2.9
Aging PP	0.50	16.0

## Data Availability

The original data presented in the study are included in the article; further inquiries can be directed to the corresponding author.
